# *MdGGT1* Impacts Apple miR156 Precursor Levels via Ontogenetic Changes in Subcellular Glutathione Homeostasis

**DOI:** 10.3389/fpls.2019.00994

**Published:** 2019-07-31

**Authors:** Yakun Chen, Qingbo Zheng, Xiaolin Jia, Keqin Chen, Yi Wang, Ting Wu, Xuefeng Xu, Zhenhai Han, Zhihong Zhang, Xinzhong Zhang

**Affiliations:** ^1^College of Horticulture, China Agricultural University, Beijing, China; ^2^Horticulture College, Shenyang Agricultural University, Liaoning, China

**Keywords:** glutathione, γ-glutamyl transpeptidase, *Malus domestica* Borkh., microRNA, miR156, thiol redox, vegetative phase change

## Abstract

The vegetative phase change in flowering plants is controlled by microRNA156 (miR156) under transcriptional regulation. However, the developmental signals upstream of miR156 are not well understood. The glutathione/glutathione disulfide (GSH/GSSG) ratios and GSH levels decline significantly during phase change, which is consistent with miR156 expression in apple (*Malus domestica* Borkh.). Here, we found that the content of protein conjugated glutathione was remarkably higher in chloroplasts and nuclei of adult than juvenile phase apple hybrids. The decrease in miR156 expression was most relevant to the activities of serine acetyltransferase (SAT) and soluble γ-glutamyl transpeptidase (GGT), and the expressions of *MdGGT1* or *MdSATs*. Transgenic apples over-expressing *MdMIR156* or miR156-mimetic (MIM156) did not alter *MdGGT1* expression or the soluble GGT activity. Inhibition of GGT activity with serine-borate complex or acivicin led to significant reduction in GSH content, the GSH/GSSG ratio, and the expressions of *MdMIR156a5*, *MdMIR156a12*, and miR156. Depletion of GSH with diethyl maleate without altering GGT activity caused a dramatic decrease in the expression of *MdMIR156a5*, *MdMIR156a12*, and miR156. Manipulating GGT activity and GSH homeostasis by transgenic over-expressing or RNAi *MdGGT1* increased or decreased *MdMIR156a5* and *MdMIR156a12* levels, respectively. These data provided novel evidence that *MdGGT1* participates in transcriptional level of transcription regulation of miR156 precursors during ontogenesis.

**HIGHLIGHTS:**

- MdGGT1 affects thiol redox status and indirectly participates in the regulation of miR156 expression during vegetative phase change.

## Introduction

Flowering is a critical life-history trait for the reproductive success of plants (Wang et al., 2009; Wang, 2014; Wei et al., 2017). The initiation of flowering is controlled by both environmental cues and endogenous regulation (Zhou et al., 2013). Phase change from the juvenile to the adult vegetative phase is a typical endogenous pathway (Poethig, 1990; Poethig, 2003). microRNA156 (miR156) has been identified as a key factor regulating phase change and is highly conserved in the plant kingdom (Carrington and Ambros, 2003; Wu and Poethig, 2006; Chuck et al., 2007; Wang et al., 2009, 2011; Xie et al., 2012; Bergonzi et al., 2013; Yoshikawa et al., 2013; Zhou and Wang, 2013).

miR156 is under transcriptional regulation. In *Arabidopsis*, the expression level of miR156 is attributed to the transcriptional level of the precursor genes, *MIR156A* and *MIR156C* (Yang et al., 2013). The double stranded RNA-binding protein HYPONASTIC LEAVES1 (HYL1) is required for the processing of mature miR156 from its primary transcripts pri-miR156a, *HYL1* expression in adult *Arabidopsis* plants increased as over three times higher than its juvenile phase, but the mature miR156 decreased significantly in adult phase, showing that miR156 is mainly regulated under transcription level (Li et al., 2012). In the apple (*Malus domestica* Borkh.) genome, seven putative *MdMIR156* genes were dominantly expressed and the transcription of *MdMIR156a5* and *MdMIR156a12* decreased significantly in leaves and shoot tips, respectively, during the vegetative phase change, which was consistent with the changes in mature miR156 level (Jia et al., 2017). No substantial changes were detected in *MdHYL1* expression during the vegetative phase change (Jia et al., 2017). The transcription levels of pre-miR156 were regulated by upstream signals derived from the leaf primordia in *Arabidopsis* (Yang et al., 2011). Although sugar promotes vegetative phase change in *A. thaliana* by repressing the expression of miR156, sugar is likely not the unique upstream signal in perennials (Proveniers, 2013; Yang et al., 2011, 2013; Yu S. et al., 2013). The *Arabidopsis* mutant *gin2-1*, which lacks HEXOKINASE 1 (HXK1), is only slightly precocious in the transition to the adult phase, which indicates that sugar may not be the only factor that regulates miR156 expression (Yang et al., 2013).

**FIGURE 1:**
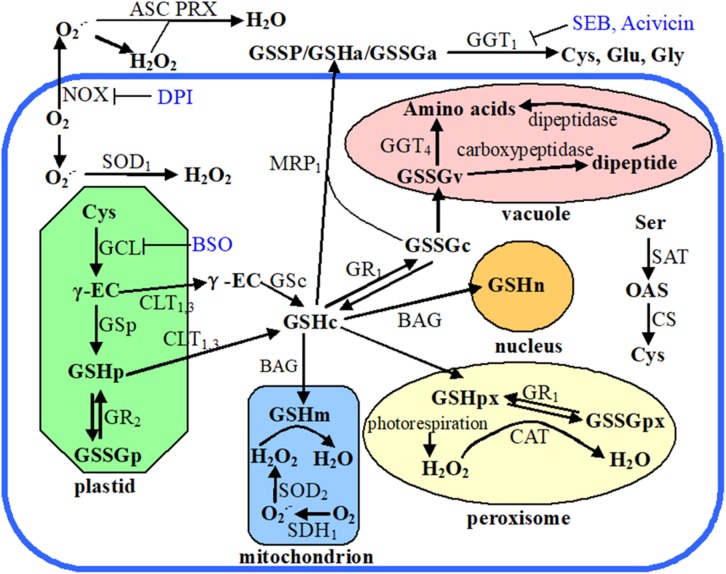
Schematic diagram showing plant cellular glutathione metabolism, recycling, and compartmentalization. Endogenous chemicals are presented in bold font, enzymes and transporters are in nonbold font, while exogenous inhibitors are in blue colored font. The subscript letters following the chemical abbreviations indicate the subcellular localizations: apoplast (a), cytosol (c), plastid (p), mitochondrion (m), peroxisome (px), nucleus (n), and vacuole (v). Organelles are marked with different color, the thick blue open rectangle means plasma membrane, and outside of that rectangle indicates apoplast. Abbreviations used in this figure: ASC, ascorbate; Acivicin, α-amino-3-chloro-4,5-dihydro-5-isoxazoleacetic acid; BAG, BCL2-associated athanogene; BSO, buthionine sulfoximine; CAT, catalase; CLT, chloroquine resistance-like transporter; CS, O-acetylserine sulfhydrylase; Cys, cysteine; DPI, diphenyleneiodonium; γ-EC, γ-glutamylcysteine; GCL, glutamate cysteine ligase; GGT, γ-glutamyl transpeptidase; Glu, glutamate; Gly, glycine; GR, glutathione reductase; GS, glutathione synthetase; GSH, glutathione; GSSG, glutathione disulfide; GSSP, S-glutathionylated protein; MRP, multidrug resistance protein; NOX, NADPH oxidase; OAS, O-acetylserine; PRX, peroxiredoxin; SAT, serine acetyltransferase; SDH, succinate dehydrogenase; SEB, serine-borate complex; Ser, serine; SOD, superoxide dismutase.

Age-associated changes in reactive oxygen species (ROS) and antioxidant levels have been found in many species, including *Drosophila melanogaster*, *Musca domestica*, and mice (*Mus musculus*) (Cochemé et al., 2011; Sohal and Orr, 2012). During the phase change in apple, the content of H_2_O_2_ increased remarkably, which is not robustly consistent with the decrease in miR156 expression (Du et al., 2015; Jia et al., 2017). Glutathione (γ-glutamyl-cysteinyl-glycine) is an essential metabolite with multiple functions in plants (Noctor et al., 2012). Reduced glutathione (GSH) is continuously oxidized to a disulfide form (GSSG) that is recycled to GSH by NADPH-dependent glutathione reductase (GR) (Figure 1). Glutathione/glutathione disulfide (GSH/GSSG) ratios declined in the brain tissue of aging mice compared to young mice (Rebrin et al., 2007). Identically, the decline of GSH/GSSG ratio during aging has also been reported in *Caenorhabditis elegans* (Brys et al., 2007). Similarly, in apple, we found that the GSH content and the GSH/GSSH ratio decreased significantly during ontogenesis, which is consistent with the changes in the expressions of *MdMIR156a5*, *MdMIR156a12*, and mature miR156 (Du et al., 2015; Jia et al., 2017). Notably, when redox homeostasis was altered by exogenous L-2-oxothiazolidine-4-carboxylic acid or buthionine sulphoximine treatments *in vitro*, *MdMIR156a5*, *MdMIR156a12*, and mature miR156 expressions changed correlated with GSH levels and the GSH/GSSG ratio but did not correlate with H_2_O_2_ content (Du et al., 2015; Jia et al., 2017). No substantial differences in the content of H_2_O_2_ and GSH were observed between the transgenic *Nicotiana benthamiana* lines *MdMIR156a6-*overexpressing, miR156-mimetic (MIM156), and the untransformed wild type (Jia et al., 2017). These results implied that the regulation of GSH is upstream of *MdMIR156s*.

Cellular GSH potential is precisely controlled via its biosynthesis, subcellular compartmentalization, catabolism, and reutilization of the oxidized products (Figure 1; Noctor et al., 2012). The rate-limiting reaction of glutathione biosynthesis is catalyzed by glutamate-cysteine ligase (GCL) (Hicks et al., 2007). Besides GCL activity, cysteine (Cys) availability is another dominant factor affecting glutathione biosynthesis and thus regulating cellular GSH homeostasis (Noctor et al., 2012). Cys availability is determined by both the *de novo* synthesis of Cys and Cys recycling. The *de novo* biosynthesis of Cys is catalyzed by cysteine synthase (CS) and serine acetyltransferase (SAT) (Figure 1; Wirtz and Hell, 2007). Gamma (γ)-glutamyl transpeptidase (GGT) enzymes are responsible for the breakdown of GSSG or S-glutathionylated proteins and provide free cysteine residue for reutilization, thereby enabling the cells to maintain their intracellular GSH levels (Martin et al., 2007; Hanigan, 2014). GGT is the only enzyme that is capable of catalyzing the hydrolysis of the unique N-terminal amide bond between glutamate and cysteine (Martin and Slovin, 2000). Of the four *GGT* genes in *Arabidopsis*, GGT1 and GGT2 are localized at the apoplast side of the plasma membrane, GGT3 is a pseudogene, and GGT4 is localized in the vacuole (Figure 1; Grzam et al., 2007; Martin et al., 2007; Ohkama-Ohtsu et al., 2007; Gigolashvili and Kopriva, 2014). In *Arabidopsis*, GGT1 accounts for 80–90% of GGT activity in all tissue except seeds and GGT1 activity is sensitive to the apoplast redox environment (Martin et al., 2007; Zhang and Forman, 2009). *GGT1* knockout mutant protoplasts of *A. thaliana* are unable to retrieve GSH from the growth medium, indicating the importance of GGT for GSH recycling (Ohkama-Ohtsu et al., 2007).

To explore if and how cellular GSH homeostasis regulates *MdMIR156s* levels and subsequently controls the vegetative phase change, we used apple hybrid trees grown from seeds as a model for the investigation of phase change in woody perennials. We found that *MdGGT1* played an important role in the regulation of miR156 transcription during ontogenesis.

## Materials and Methods

### Plant Materials and Chemical Treatments

Three 9 years old trees raised from hybrid seeds of the cross *Malus asiatica* “Zisai Pearl” × *M. domestica* “Red Fuji” were used as three biological replicates in this study. “Zisai Pearl” is a Chinese domestic cultivar originating from Hebei. On early May, 2015, newly expanded young leaves and unlignified shoot tips were collected from 1 year old suckers on the trunk and annual branches from the canopy top, immediately put into liquid nitrogen and stored in −80°C refrigerator. Since the discovery of miR156 to regulate vegetative phase change, miR156 is now extensively used as molecular marker to determine juvenile phase. By using miR156 marker, the samples taken from the 1st to the 80th nodes were defined as the juvenile phase (J), and those from the 120th node to the canopy top were defined as the adult phase (A) (Du et al., 2015; Jia et al., 2017).

Tissue cultured micro-shoots were used for exogenous reagent treatments; those micro-shoots were derived from juvenile phase shoot tip meristem and sub-cultured by nodal stem segments on Murashige and Skoog (MS) media. The redox modulating reagents were added to the culture media after filtration sterilization. GGT activity can be impaired by the competitive inhibitor serine-borate complex (SEB) (S4500/B3545, Sigma-Aldrich, St. Louis, MO, United States) and the noncompetitive α-amino-3-chloro-4,5-dihydro-5-isoxazoleacetic acid (acivicin) (A2295, Sigma-Aldrich, St. Louis, MO, United States) (Dringen et al., 1997; Ferretti et al., 2008). Here, 10 mM SEB and 50 μM acivicin were used as the competitive and noncompetitive GGT inhibitors, respectively (Stole et al., 1994; Destro et al., 2011). The micro-shoots were treated by adding the abovementioned GGT inhibitors to the culture media. Ten milli molar diethyl maleate (DEM) (D97703, Sigma-Aldrich, St. Louis, MO, United States) was used as a GSH depletor. The concentrations used for DEM treatments were tested in preliminary experiments (Supplementary Figure S1).

### Chloroplast Fractionation

Chloroplasts were isolated according to the protocols described by Preuss et al. (2014). Leaves were collected and chopped finely with a razor blade in homogenization buffer (0.45 M sorbitol, 20 mM Tricine:KOH pH 8.4, 10 mM ethylene diamine tetraacetic acid (EDTA), 10 mM NaHCO_3_, 0.1% (w/v) fatty acid-free bovine serum albumin (BSA), and 1× protease inhibitors). The mixture was filtered through cheesecloth and spun at 1,500 × *g* at 4°C. Pelleted chloroplasts were suspended in resuspension buffer (0.3 M sorbitol, 20 mM Tricine: KOH pH 7.6, 5 mM MgCl_2_, and 2.5 mM EDTA), layered over a preformed 40% Percoll gradient, and centrifuged at 5,000 × *g* (4°C) with ultracentrifuge with a swing-bucket rotor. A thin band of intact chloroplasts were collected, diluted in resuspension buffer, and centrifuged again at 3,500 × *g* (4°C). The pellet was resuspended again in resuspension buffer. The chloroplast suspension was then kept on ice in the dark until further use.

### Nuclei Fractionation

To isolate nuclei, leaves were harvested and homogenized in the nuclear isolation buffer at pH 6.0 (10 mM Mes-KOH, 10 mM NaCl, 5 mM EDTA, 0. 15 mM spermine, 0.5 mM spermidine, 0.6% Triton X-100, 0.25 M Sucrose, pH 6.0) with a blender equipped with razor blades. The crude homogenate was then filtered through stainless steel mesh and the nuclei in the filtrate were collected by centrifugation at 1,500 × *g*. The nuclei were further purified twice on 0–50% discontinuous Percoll gradient (Sakamoto and Nagatani, 1996). The nuclei pellet was finally resuspended in 60 mM Hepes, 6 mM MgSO_4_, 20% glycerol, and 10 mM 2-mercaptoethano1, pH 7.0.

### Mitochondria Fractionation

Mitochondria were isolated using the method described by Keech et al. (2005). Leaves were ground finely with a razor blade in homogenization buffer (0.3 M sucrose, 10 mM N-tris (hydroxymethyl)-methyl-2-2aminoethanesulphonic, 2 mM EDTA, 10 mM KH_2_PO_4_, 25 mM tetrasodium pyrophosphate, 1 mM glycine, 1% (w/v) polyvinylpyrrolidone-40, 1%(w/v) defatted BSA, 50 mM sodium ascorbate, 20 mM cysteine, pH-(KOH) 8.0), then filtered through 20 um nylon mesh. The extract was centrifuged at 2,500 × *g* for 5 min and the resulting supernatant was centrifuged at 15,000 *g* for 15 min. The pellet obtained was suspended in 0.5–1.0 mL of wash buffer [0.3 M sucrose, 10 mM TES, 10 mM KH_2_PO_4_, pH-(KOH) 7.5] and very gently homogenized twice, using a chilled glass homogenizer. A 50% Percoll gradient was centrifuged at 39,000 *g* for 40 min in ultracentrifuge with a swing-bucket rotor. The crude mitochondrial fraction was carefully layered on top of preformed Percoll gradients. After centrifugation at 15,000 *g* for 15 min, the mitochondria formed a whitish band close to the bottom of the tube. The pellet was resuspended in 0.3 M sucrose, 10 mM TES, and 10 mM KH_2_PO_4_, pH-(KOH) 7.5.

### Analysis of Glutathione Homeostasis and Enzyme Activity

For tissue samples, free glutathione (the sum of GSH and GSSG levels) was quantified using a GSH and GSSG assay kit (Beyotime, Nantong, China) following the manufacturer’s instructions. The measurements of GSH and GSSG were monitored by a microplate reader (Model 680, Bio-Rad, Hercules, CA, United States). The GSH/GSSH ratio was then calculated. For subcellular compartments, because it’s difficult to preserve glutathione pool against oxidation during the conventional extraction and purification procedures required to obtain subcellular fractions, the absolute concentrations of free glutathione are not possible to be precisely determined (García-Giménez et al., 2013; Noctor et al., 2016), the subcellular levels of free glutathione per gram organelle protein were measured as ratio of A relative to J by using the thiol-group reaction strategy with a GSH and GSSG assay kit (Beyotime) (Galkina et al., 2017). The isolation of soluble and cell wall conjugated GGT was described by Destro et al. (2011). The activity of GCL and GGT was determined using a GCL assay kit (Jian Cheng, Nanjing, China) and a GGT assay kit (Solarbio, Beijing, China), respectively. The assay of SAT and CS activity was conducted according to Watanabe et al. (2008) and Nguyen et al. (2012), respectively.

### Transmission Electron Microscopy and Immunogold Labeling

Ultrathin sections were prepared and immunogold labeled according to the protocol described by Zechmann and Müller (2010). Briefly, the ultrathin sections from J and A leaves were initially incubated with the primary antibody (anti-glutathione rabbit polyclonal IgG, Agrisera Corp., Vännäs, Sweden) at a dilution of 1:3,000 in the dilution buffer. This antibody recognizes only the conjugated glutathione and GSH but not GSSG. Then the ultrathin sections were treated with secondary antibody (goat anti-rabbit IgG antibody conjugated with 10 nm gold, Sigma-Aldrich, St. Louis, MO, United States) at a dilution of 1:20. The sections were finally double-stained with uranyl acetate-lead citrate and examined with a JEM-100S electron microscope (JEOL, Tokyo, Japan). Micrographs of randomly photographed immunogold labeled sections were digitized with computer aided drafting software and gold particles were manually counted. A minimum of at least 10 different cells were analyzed for gold particle density per sample. The gold particle density data in the organelles were normalized against the cytosolic background.

### Free Cysteine Quantification

Free cysteine was quantified using the method described by Samara et al. (2016) with minor modifications. Briefly, leaves were harvested and homogenized in isolation buffer at pH 8.0 (10 mM EDTA, 180 mM borate), and centrifuged at 1,118 g for 5 min. The supernatants were filtered through 0.45 μm membrane filters. A 200 μL volume of the treated sample was subsequently subjected to derivatization; 200 μL of a standard cysteine-mixture (or sample), 100 μL of borate buffer (200 mmol L-1, pH = 8.0), 100 μL of methyl propiolate (200 mmol L^–1^), and 600 μL of EDTA solution (5 mmol L^–1^) were added to a heavy-wall borosilicate micro-reaction vial. The sample was then manually mixed and the reaction was left to proceed at room temperature for 10 min while protected from light. Cys was determined by a high performance liquid chromatography system (Waters1525, MA, United States) equipped with a Waters 2487 Dual Absorbance Detector. The thioacrylates of Cys were eluted in the isocratic mode, with a mobile phase of 15 mM (NH_4_)_2_HPO_4_/H_3_PO_4_ (pH = 2.2)/methanol (92:8v/v), containing 1 mM EDTA. The flow rate was 0.2 mL min^–1^, and the injection volume was 2 μL. Detection was carried out at 285 nm. Cys was identified by its retention time and was quantified using an external standard.

### Cloning of Apple *MdGGT*s

*MdGGT* genes in the apple genome were searched on the Rosaceae genome database^1^ and GenBank^2^. Primers designed from the cDNA sequence of apple are shown in Supplementary Table S1. Total RNA was prepared from apple tissue and both RT-PCR and PCR were performed as previously described (Zhang et al., 2004).

### Relative Expression Assay

Total RNA were extracted using a modified cetyltrimethylammonium bromide method followed by DNase I digestion to remove any DNA contamination (TaKaRa Biotechnology Co., Ltd.) (Gasic et al., 2004). Complementary DNA was synthesized from total RNA using a cDNA Synthesis Kit (Takara)^3^. Quantitative RT-PCR were performed using SYBR green reagents (RR820A, Takara, Dalian, China) in an Applied Biosystems 7500 real-time PCR system. MicroRNA was extracted by using the RNAiso for Small RNA kit (9753Q, Takara, Dalian, China) according to the manufacturer’s instruction. For reverse transcription of miRNA, 2 μg miRNA extract was diluted in 12 μL diethyl pyrocarbonate (DEPC) water plus 1 μL of dNTPs (10 mmol L^–1^, Takara, Dalian, China) and 1 μL stem-loop primer (10 μM). The mixture was incubated at 70°C for 5 min, and then immediately put on ice for 5 min. Afterward, 1.25 μL dNTPs (10 mmol L^–1^, Takara), 2.5 μL buffer (5×) (Takara), 0.6 μL RNasin, and 1 μL M-MLV (5 U μL^–1^, Takara) were added to the extracted solution. Reverse transcription was accomplished at 42°C for 1 h, followed by 70°C for 10 min. miR156 expression level was analyzed by qRT-PCR with an AB7500 Real-time PCR System and the SYBR Green fluorescence dye (FP401, TianGen, Beijing, China) (Xiao et al., 2014).

The sequences of the primer pairs are listed in Supplementary Tables S2, S3. Relative expression levels were calculated according to the 2^–ΔΔ*ct*^ method (Livak and Schmittgen, 2001).

### Subcellular Localization of MdGGT-GFP

The Super promoter: MdGGT-GFP construct was made as follows. The full-length MdGGT cDNA was fused to the upstream of the green fluorescent protein (GFP) between the SwaI (5′end)/KpnI (3′ end) sites in pCAMBIA1300-GFP vector (3560628, BioVector, China) using primers: 5′-ATTTAAATATGGGGGAGCAGAGCTTGGAA-3′ (forward) and 5′-GGGGTACCTCATACGGCTGCAGGCCTC-3′(reverse).

Onion epidermal cells were extracted from onion bulbs and cultured in MS media for 6 h at 22°C. Using the PDS-1000 particle delivery system (Bio-Rad, CA, United States), the fusion constructs were introduced through gold particles into the epidermal cells of onion bulbs; onion epidermal cells were cultured in the dark at 22°C for 24 h. GFP fluorescent signals were examined with a confocal laser-scanning microscope (Zeiss LSM510 META, Oberkochen, Germany) in the 488 nm excitation wavelength.

### Plant Transformation

*MdMIR156a6* (MDC018927.245) was chosen for miR156 precursor overexpressing, and artificial target mimics were generated by modifying the sequence of the AtIPS1 gene to knock-down miR156 expression (Xu et al., 2017). The construction of *MdMIR156a6* and MIM156 was provided by Dr. Xu (Xu et al., 2017). The two constructs were cloned behind the constitutive CaMV 35S promoter and upstream of β-glucuronidase (GUS) gene in the pBI121 vector with GUS. The RNAi vector was offered by Shenyang Agricultural University. It was constructed by inserting the intron fragment from pKANNIBAL vector into the multiple cloning site of a plant overexpression vector-pRI 101-AN (Song et al., 2017). For constructing the 35S::*MdGGT* RNAi vector, a 351 bp *MdGGT* fragment was amplified. The pRNAi -MdGGT vector was constructed by inserting the 351 bp forward and reverse fragments of MdGGT into pRNAi-E. The overexpression construct for *MdGGT* was made by cloning the full-length cDNA into the pRI 101-AN vector downstream of the 35S promoter. Primer sequences are listed in Supplementary Table S4. Transformation of apple was conducted by *Agrobacterium*-mediated transformation system using *in vitro* cultured leaflets of GL-3 as explants (Dai et al., 2013).

### GUS Staining

OEMdMIR156 and MIM156 transgenic apple were identified by GUS staining (Supplementary Figure S2). Histochemical observations of the OEMdMIR156 and MIM156 transgenic apple were performed by incubating the plant in GUS-staining solution [100 mm phosphate buffer (pH 7.5), 1 mm 5-bromo-4-chloro-3-indoylglucuronide (X-Gluc) dissolved in N,N-dimethylformamide, 10 mm Na_2_-EDTA, 1 mm potassium ferricyanide, 1 mm potassium ferrocyanide and 0.1% Triton X-100] at 37°C overnight. Stained samples were cleaned with 80% ethanol and were observed (Supplementary Figure S2).

### DNA Identification of Transgenic Plant

Transgenic apple were identified by DNA-PCR identification. Extraction of genomic DNA was used CTAB methods (Gasic et al., 2004). Sequences of the primer pairs are listed in Supplementary Table S5.

### Statistical Analysis

This experiment was a randomized block design conducted on a single plot with three replicates per treatment. All data are presented as means ± SE of each treatment and tested with repeated measures ANOVA, followed by least significant difference (LSD) tests or Duncan’s multiple-range test Significance was set at *P* < 0.05.

## Results

### Changes in Subcellular GSH Homeostasis

**FIGURE 2:**
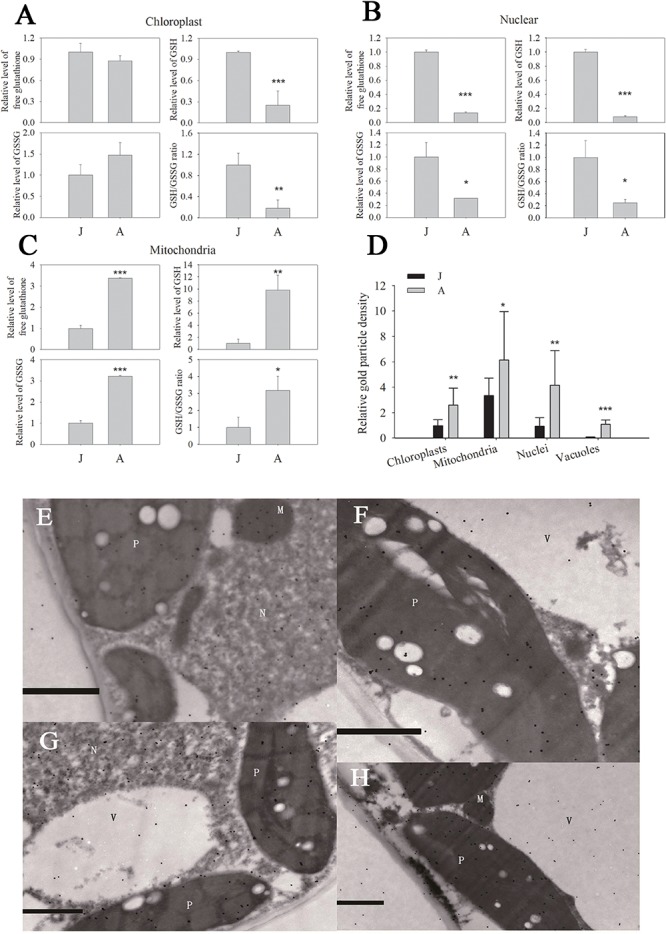
Free glutathione **(A–C)** and protein conjugated **(D)** glutathione in organelles of leaves in juvenile (J) and adult (A) phase of apple hybrids (*Malus asiatica* “Zisai Pearl” × *M. domestica* “Red Fuji”). Transmission electron micrographs showed the subcellular distribution of glutathione in leaves in the juvenile **(E,F)** and adult **(G,H)** phase. Gold particles bound to glutathione could be found in plastids (P), mitochondria (M), nuclei (N), and vacuoles (V). The gold particle density in the organelles shown in **(D)** was the relative value normalized against which in the cytosol. “^*^”, “^∗∗^” and “^∗∗∗^” indicate significant difference at *P* < 0.05, *P* < 0.01, and *P* < 0.001 (LSD tests), respectively. Error bars in **(A–C)** represent the standard deviations of three replicates. Bars in **(E–H)** indicate 1.0 μm. N > 10 in **(D)**.

We previously reported that the GSH level and GSH/GSSG ratio declined significantly during ontogenesis in apple seedlings (Du et al., 2015; Jia et al., 2017). In chloroplast fractions, the free glutathione (the sum of GSH and GSSG levels), GSH and GSSG levels of A were 0.8740, 0.2480, and 1.4735 times J (Figure 2A). Similarly, free glutathione in nuclei decreased by 86.1% in A leaves compared to J. GSH and GSSG levels of nuclei in A leaves also declined dramatically (Figure 2B). However, free glutathione level in mitochondria increased by 236.7% in A compared to in J leaves (Figure 2C), and the increase in total GSH content in mitochondria was attributed to GSSG (Figure 2C). In chloroplasts and nuclei, the GSH/GSSG ratios in J leaves were considerably lower than in A leaves (Figures 2A,B). The GSH/GSSG ratio of leaves also increased in mitochondria during ontogenesis (Figure 2C).

The subcellular distribution of protein conjugated glutathione and GSH in apple leaves was visualized by immunogold particles. Gold particles were found in plastids, mitochondria, nuclei, and vacuoles (Figures 2E–H). The relative immunogold density was significantly higher in these organelles of A than that of J leaves (Figure 2D). Since the GSH level was lower in chloroplasts and nuclei of A than J leaves, the density of immunogold was attributed to the protein conjugated glutathione. These data indicated that the levels of free glutathione, GSH, and the ratio of GSH/GSSG were significantly lower in chloroplasts and nuclei but the protein conjugated glutathione content was remarkably higher in A than in J leaves.

### The Correlation Between miR156 Expression and Glutathione Homeostasis

**FIGURE 3:**
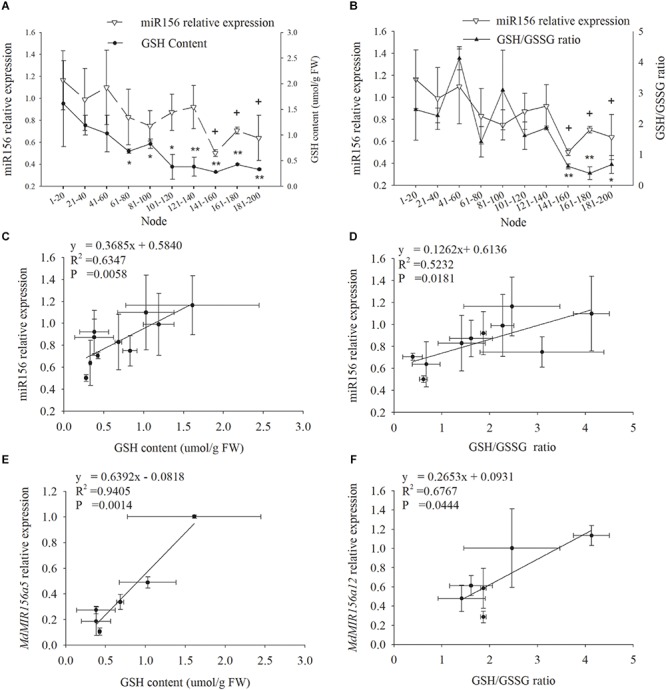
Dynamic changes in miR156 expression, GSH contents and GSH/GSSG ratio in leaves during ontogenesis of apple hybrids (*Malus asiatica* “Zisai Pearl” × *M. domestica* “Red Fuji”) **(A,B)** and the correlations between miR156 and its precursor genes expressions and GSH content or GSH/GSSG ratio **(C–F)**. Error bars represent the standard deviations of three biological replicates. “^*^” and “^∗∗^”” indicate significant difference at *P* < 0.05, *P* < 0.01, respectively, in GSH content and GSH/GSSG ratio. “+” indicate significant difference at *P* < 0.05 in miR156 relative expression.

miR156 expression declined in leaf samples during ontogenesis in accordance with GSH and the GSH/GSSG ratio (Figures 3A,B). Significant positive correlation was exhibited between miR156 expression and GSH (*P* = 0.0058) or the GSH/GSSG ratio (*P* = 0.0181) (Figures 3C,D). The expression of *MdMIR156a5* was significantly positively correlated with GSH content, and the expression of *MdMIR156a12* was significantly positively correlated with the GSH/GSSG ratio (Figures 3E,F). The correlations between *MdMIR156a5* expression and GSH/GSSG ratio, and between *MdMIR156a12* expression and GSH level were also positive, but were not statistically significant due to large standard deviations (data not shown).

**FIGURE 4:**
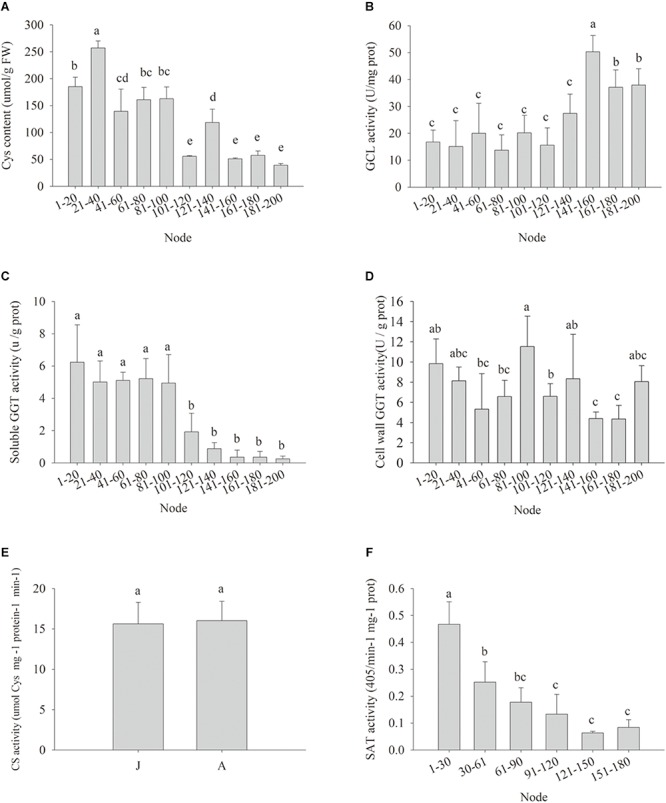
Changes in cysteine contents (Cys) **(A)**, enzyme activity of glutamate-cysteine ligase (GCL) **(B)**, soluble γ-glutamyl transpeptidase (GGT) **(C)**, cell wall conjugated GGT **(D)**, cysteine synthase (CS) **(E),** and serine acetyltransferase (SAT) **(F)** in leaves during ontogenesis of apple hybrids (*Malus asiatica* “Zisai Pearl” × *M. domestica* “Red Fuji”). Error bars represent the standard deviations of three biological replicates. The different lower-case letters above each column indicate the statistical significance (*p* < 0.05) by analysis of variance followed by Duncan’s multiple-range test.

Cellular Cys availability may affect GSH *de novo* biosynthesis (Noctor et al., 2012). The Cys content in the leaf samples declined gradually during ontogenesis, and the cutoff for significant decrease appeared at nodes 101–120 (Figure 4A), implying the biosynthesis of GSH might be restricted by Cys availability, despite the GCL activity was abruptly and significantly increased from nodes 141 to 160 (Figure 4B). The possible causes of reduced Cys content could be less Cys *de novo* synthesis and/or Cys recycling. The soluble GGT activity decreased significantly at nodes 101–120, but the activity of cell wall conjugated GGT showed no obvious changes at different nodes (Figures 4C,D). No significant difference was detected in CS enzyme activity between J and A leaves (Figure 4E), but the activity of SAT, another enzyme for plant Cys biosynthesis, decreased gradually with node numbers (Figure 4F). Consistently, the expressions of the five *MdSAT* gene members were significantly lower in A than J samples (Figure 4E).

**FIGURE 5:**
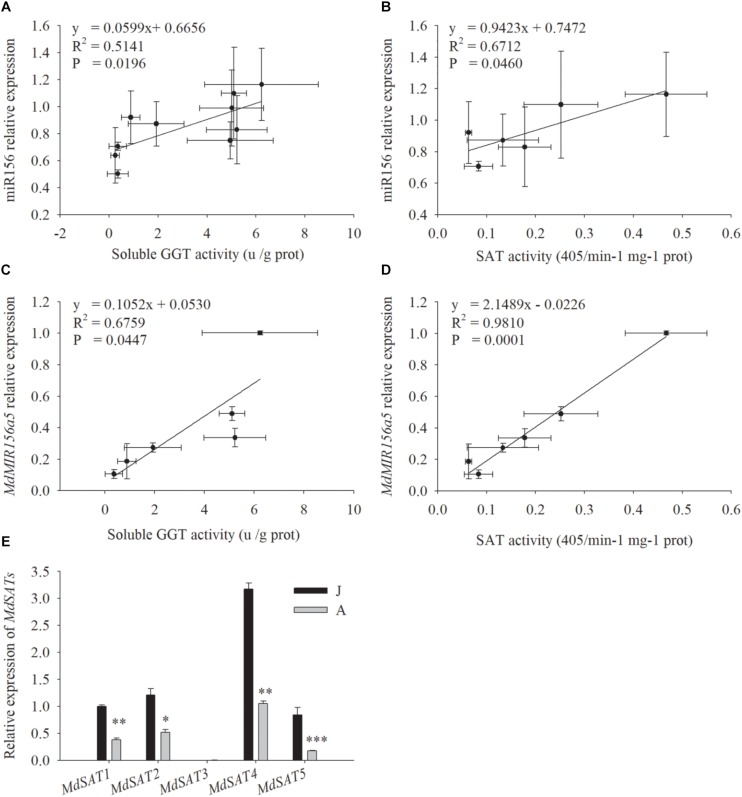
Correlations between relative expressions of miR156, *MdMIR156a5* and the activity of soluble GGT and SAT **(A–D)**, and relative expressions of *MdSATs* **(E)** in leaves during ontogenesis of apple hybrids (*Malus asiatica* “Zisai Pearl” × *M. domestica* “Red Fuji”). Error bars represent the standard deviations of three biological replicates. “^*^,” “^∗∗^,” and “^∗∗∗^” indicate significant difference (LSD tests) at *P* < 0.05, *P* < 0.01, and *P* < 0.001, respectively.

miR156 expression was correlated with both SAT enzyme activity (Figure 5B, *P* = 0.046) and soluble GGT activity (Figure 5A, *P* = 0.0196) (Figures 5A,B). The expression of *MdMIR156a5*, which is the most actively expressed miR156 precursor gene in leaves (Jia et al., 2017), was significantly correlated with soluble GGT activity and SAT activity (Figures 5C,D). The expression of *MdMIR156a12* was also correlated with activities of GGT and SAT but the correlation coefficients were not statistically significant (Supplementary Figure S3), most possibly because *MdMIR56a12* is most abundantly expressed in shoot tips (Jia et al., 2017). Collectively, the data indicated that the decrease in miR156 expression was most relevant to soluble GGT activity and therefore the availability of recycled Cys.

### The Expression Pattern of *MdGGT*s

**FIGURE 6:**
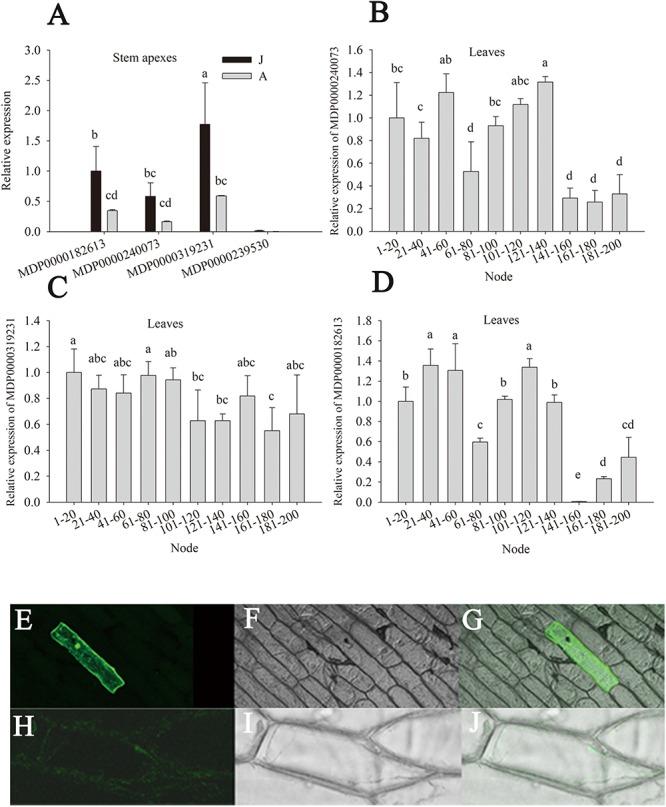
Relative expression of *MdGGTs* in the stem apex and leaves of the juvenile (J) and the adult (A) phase of apple hybrids (*Malus asiatica* “Zisai Pearl” × *M. domestica* “Red Fuji”) **(A–D)** and subcellular localization of *MdGGT1* (MDP0000319231) **(E–J)**. Transient expression of 35S: MdGGT1-GFP construct **(E–G)** and 35S: GFP construct **(H–J)** in onion epidermal cells. **(E,H)** were GFP fluorescence, **(F,I)** were transmission while **(G,J)** were merged. Error bars in **(A–D)** represent the standard deviations of three biological replicates. The different lower-case letters above each column indicate the statistical significance (*p* < 0.05) by analysis of variance followed by Duncan’s multiple-range test.

There are eight putative *MdGGT* genes in the apple genome. The expression levels of four members (MDP0000319231, MDP0000240073, MDP0000239530, and MDP0000182613) were detectable in apple stem apexes and leaves (Figure 6). Of these four *MdGGT* gene members, the expression of MDP0000239530 was lower than the other three members (Figure 6A). MDP0000182613, MDP0000240073, and especially MDP0000319231 were dominantly expressed in stem apexes or leaves. These three members exhibited much higher expression levels in J than A stem apexes, which was in accordance with the ontogenetic changes in GGT enzyme activity (Figures 6A–D). Significant decrease in leaf MDP0000319231 expression was found at 101–120 node but the expressions of leaf MDP0000182613 and MDP0000240073 were not as consistent with phase change (Figures 6B–D). The fluorescence of the 35S::GFP construct was localized predominantly in the nucleus and in the thin cytosolic layer underlying the cell membrane (Figures 6H–J), whereas GFP fluorescence of the 35S::MDP0000319231-GFP construct was observed in the whole surface of the cells (Figures 6E–G), showing the apoplast or plasma membrane localization of its coding protein. MDP0000319231 was therefore tentatively designated as *MdGGT1* according to its expression pattern, subcellular localization, and its ortholog in *Arabidopsis* (Supplementary Figures S4, S5).

No significant and robust differences in the expressions of *MdGGT1*, MDP0000182613, and MDP0000240073 were found between OEMdMIR156 and MIM156 transgenic apple lines (Supplementary Figure S2), which confirmed that the expression of *MdGGT1* was not regulated downstream of miR156 during vegetative phase change.

### The Responses of miR156 Expression to GSH Regulators

**FIGURE 7:**
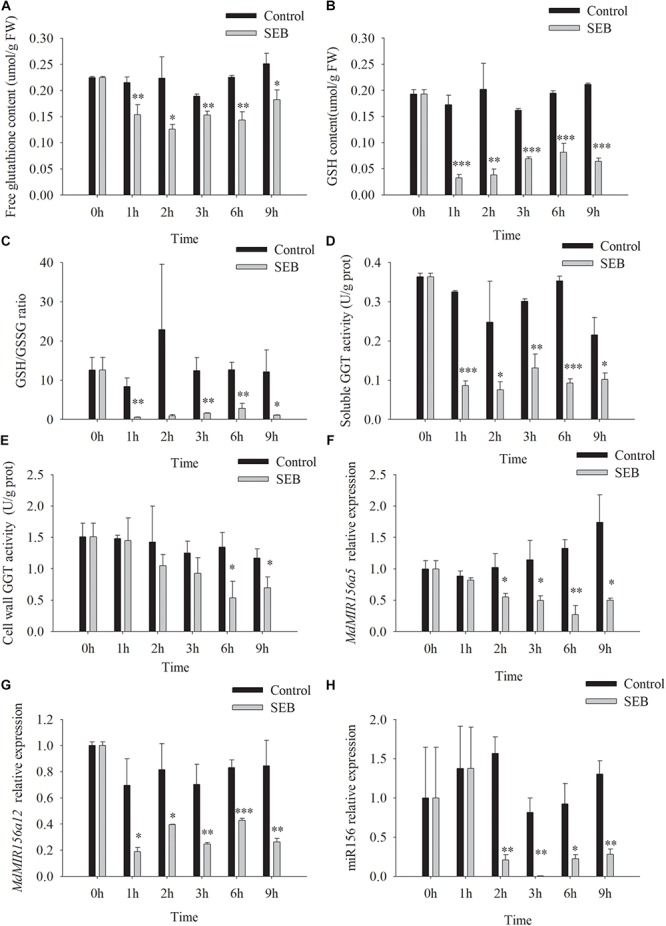
Changes in contents of free glutathione **(A)** and GSH **(B)**, GSH/GSSG ratio **(C)**, enzyme activities of soluble **(D),** and cell wall conjugated **(E)** GGT, and expressions of *MdMIR156a5* **(F)**, *MdMIR156a12* **(G),** and miR156 **(H)** in apple *in vitro* shoots after addition of 10 mM serine-borate complex (SEB) to the culture media. Error bars represent the standard deviations of three biological replicates. “^*^,” “^∗∗^,” and “^∗∗∗^” indicate significant difference at *P* < 0.05, *P* < 0.01, and *P* < 0.001, respectively.

SEB is a competitive inhibitor of GGT activity (Ferretti et al., 2008; Destro et al., 2011). After the addition of 10 mM SEB to the culture media, soluble GGT activity of apple shoots *in vitro* declined by 1 h, while cell wall conjugated GGT activity decreased significantly at 6 h (Figures 7D,E). As a result, the content of free glutathione, GSH, and the ratio of GSH/GSSG declined by 1 h after SEB treatment (Figures 7A–C). Similarly, the expression levels of *MdMIR156a12, MdMIR156a5* and mature miR156 significantly declined 1 or 2 h after SEB treatment (Figures 7F–H).

**FIGURE 8:**
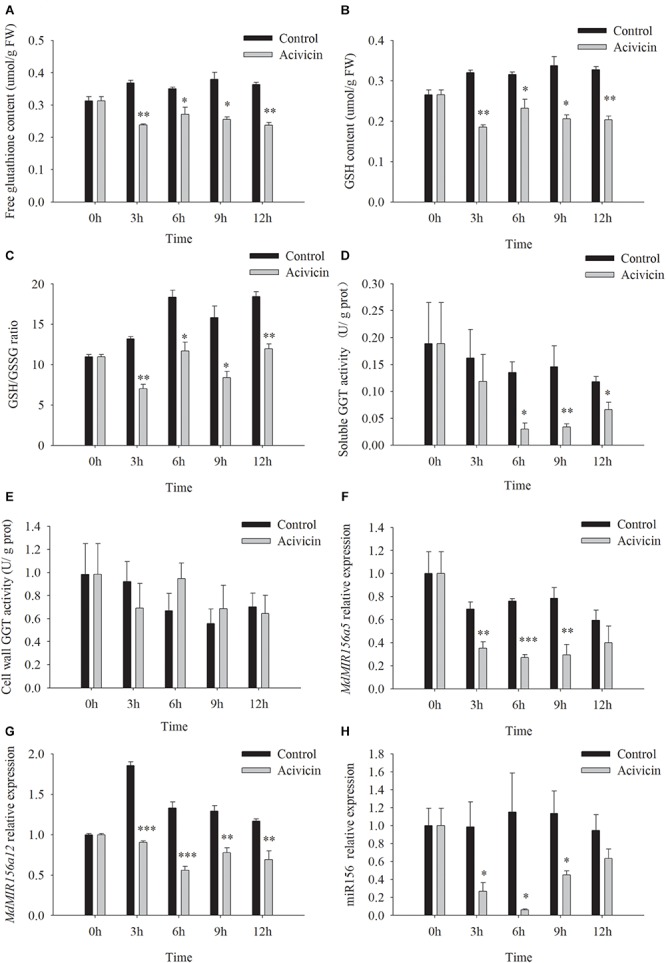
Changes in contents of free glutathione **(A)** and GSH **(B)**, GSH/GSSG ratio **(C)**, enzyme activities of soluble **(D),** and cell wall conjugated **(E)** GGT, and expressions of *MdMIR156a5* **(F)**, *MdMIR156a12* **(G)**, and miR156 **(H)** in apple *in vitro* shoots after addition of 50 μM α-amino-3-chloro-4,5-dihydro- 5-isoxazoleacetic acid (acivicin) to the culture media. Error bars represent the standard deviations of three biological replicates. “^*^,” “^∗∗^,” and “^∗∗∗^” indicate significant difference at *P* < 0.05, *P* < 0.01, and *P* < 0.001, respectively.

Unlike SEB, acivicin is a noncompetitive inhibitor of GGT activity (Stole et al., 1994; Ferretti et al., 2008; Destro et al., 2011). After the addition of 50 μM acivicin to the culture media, soluble GGT activity of apple shoots *in vitro* decreased remarkably by 26.9, 77.7, 76.8, and 43.8% at 3, 6, 9, and 12 h, respectively, but cell wall conjugated GGT activity did not change significantly with or without acivicin (Figures 8D,E). Free glutathione, GSH content, and the GSH/GSSG ratio reduced substantially by 3 h after treatment with acivicin (Figures 8A–C). As expected, the expression levels of *MdMIR156a5*, *MdMIR156a12*, and mature miR156 declined 3 h after acivicin treatment (Figures 8F–H).

When apple shoots *in vitro* were treated with 50 μM acivicin, the expression of the five highly expressed *MdSAT* gene members did not show robust decreases between timepoints or between gene members (Supplementary Figure S6), which was inconsistent with the decrease in miR156 expression (Figure 8H). These data did not support the involvement of *MdSATs* in the upstream regulation of miR156 expression.

**FIGURE 9:**
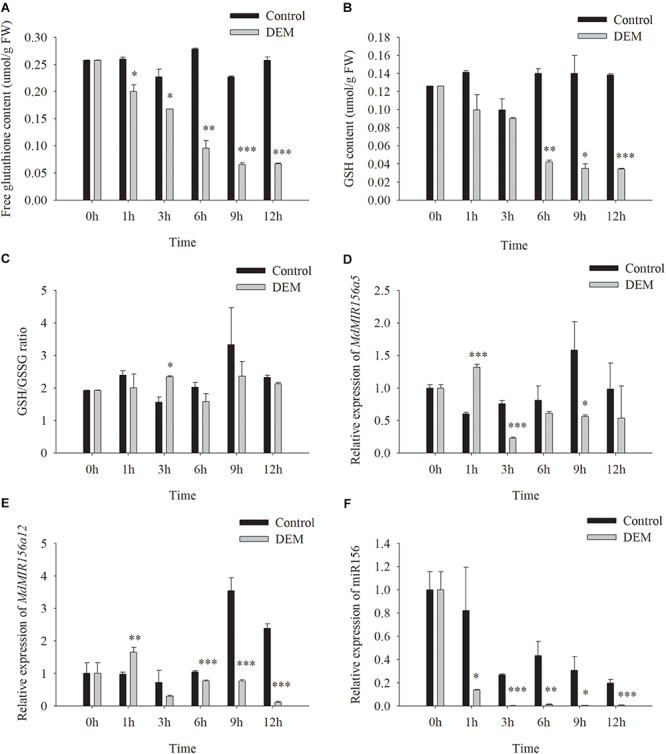
Changes in free glutathione **(A)** and GSH **(C)** contents, GSH/GSSG ratio **(E)** and the expressions of *MdMIR156a5* **(B)**, *MdMIR156a12* **(D),** and miR156 **(F)** in apple *in vitro* shoots with or without addition of 10 μM diethyl maleate (DEM) to the culture media. Error bars represent the standard deviations of three biological replicates. “^*^,” “^∗∗^,” and “^∗∗∗^” indicate significant difference (LSD test) at *P* < 0.05, *P* < 0.01, and *P* < 0.001, respectively.

DEM is a subcellular GSH depletory chemical by forming GSH-DEM adducts (Vivancos et al., 2010). Neither soluble nor cell wall conjugated GGT activity of apple shoots *in vitro* was affected by DEM treatments (Supplementary Figure S7). However, the concentrations of free glutathione and GSH were reduced 1h after the addition of 10.0 mM DEM to the culture medium (Figures 9A,B). The GSH/GSSG ratio was not altered significantly (Figure 9C). The expression levels of *MdMIR156a5* and *MdMIRa12* decrease by 3 h and mature miR156 declined immediately after DEM treatment (Figures 9D–F).

### Silencing and Overexpressing *MdGGT1* Transgenic Apple Lines

**FIGURE 10:**
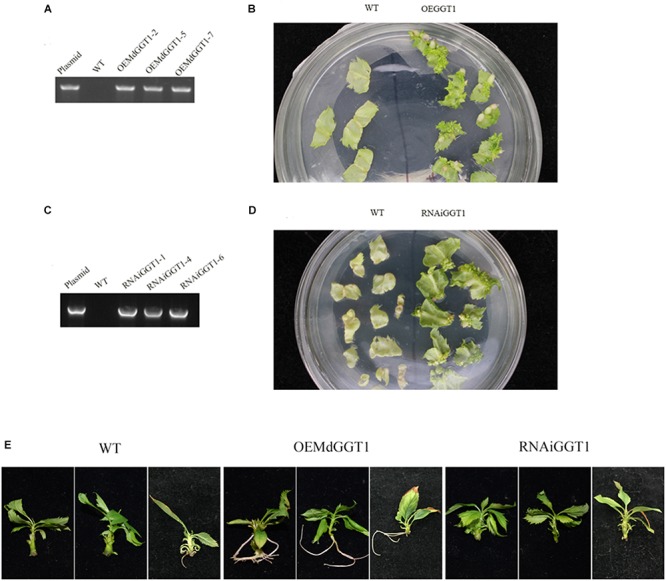
Identification and the adventitious rooting ability of apple transgenic lines over-expressing MdGGT1 **(A,B)** and MdGGT1-RNAi **(C,D)**. **(A,C)** Were PCR analysis to confirm the transformants. **(B,D)** Showed the regeneration of transformed and untransformed wild-type leaf discs with kanamycin. **(E)** Shows the differences on adventitious rooting ability between OEMdGGT1, MdGGT1-RNAi transgenic lines and untransformed wild type GL-3 (WT).

**FIGURE 11:**
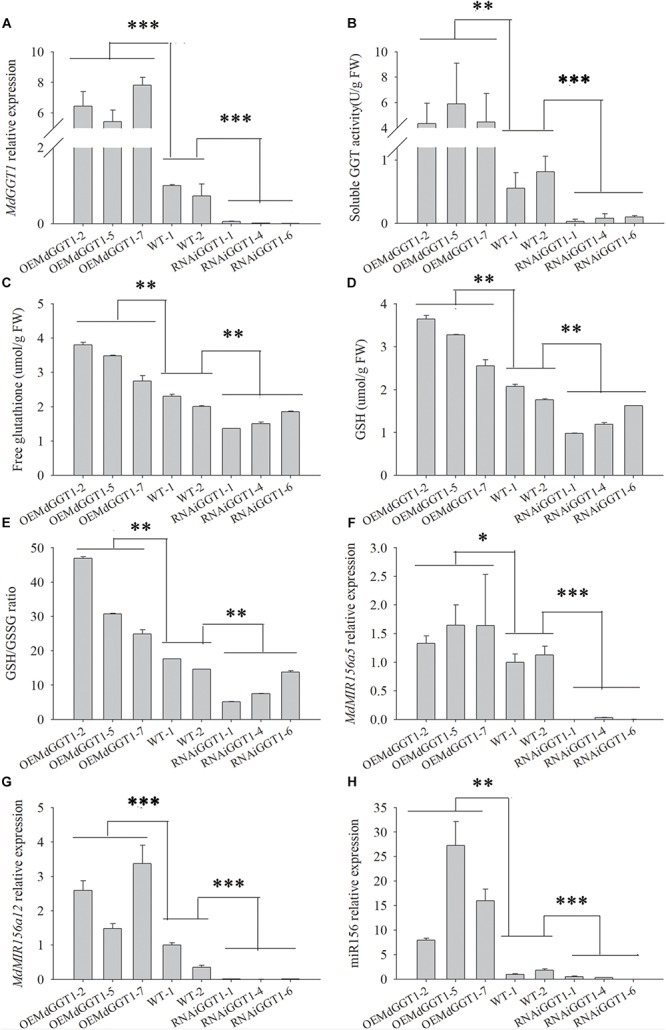
The expressions of *MdGGT1* **(A)**, soluble GGT activity **(B)**, free glutathione **(C)** and GSH **(D)** contents, GSH/GSSG ratio **(E)** and *MdMIR156a5* **(F)**, *MdMIR156a12* **(G)** and miR156 **(H)** in the plantlets of transgenic apple GL-3 lines over-expressing (OEMdGGT1) or RNAi MdGGT1, compared with untransformed control (WT). Error bars represent the standard deviations of three replicates. The different lower-case letters above each column indicate the statistical significance by analysis of variance followed by LSD multiple-range test (^*^p < 0.05; ^∗∗^*p* < 0.01; ^∗∗∗^*p* < 0.001).

Higher adventitious rooting ability is a marker trait of juvenility in many plant species (Poethig, 1990). The easiness of adventitious rooting was an observable morphological change of the OEMdGGT1 transgenic apple line (Figure 10). The number of adventitious roots that were observed 14 days after transplanting to the rooting media was 2.75 per plant in OEMdGGT1 lines, which was significantly higher than in un-transformed control (0.25 per plant) and in MdGGT1 RNAi lines (0 per plant) (Figure 10 and Supplementary Table S6). In the three apple transgenic lines overexpressing *MdGGT1*, the expression of *MdGGT1*, soluble GGT activity, free glutathione, GSH content, and the GSH/GSSG ratio were significantly higher (Figures 11A–E), and thus the expression levels of *MdMIR156a5*, *MdMIR156a12*, and mature miR156 were up-regulated compared to un-transformed wild type and MdGGT1 RNAi lines (Figures 11F–H). In contrast, the three *MdGGT1* RNAi apple lines exhibited lower free glutathione, GSH content, the GSH/GSSG ratio, and reduced expression levels of *MdMIR156a5*, *MdMIR156a12*, and mature miR156 compared with the wild type (Figure 11). Together, manipulation of GGT activity by either exogenous reagents or transgenesis could impact the transcription of *MdMIR156a5* and *MdMIR156a12*.

## Discussion

### Ontogenetic Changes in GSH Homeostasis Differed Between Subcellular Compartments

There was more GSH recruitment to the nuclei at J and relatively larger proportion of GSH was consumed in mitochondria at A (Figure 2). Similarly, the glutathione concentration varied greatly among subcellular compartments and the largest variation was found in mitochondria in *Arabidopsis* (Koffler et al., 2013). Partial depletion of nuclear GSH may impairs DNA duplication, gene expression, protein synthesis, cell proliferation, and differentiation in eukaryotes (Markovic et al., 2009; Vivancos et al., 2010; García-Giménez et al., 2013), thus the reduction of nuclear GSH should be paid much attention.

miR156 level of apple micro-shoots *in vitro* may change with GSH concentration by using exogenous redox regulatory agents, and the relative reductive thiol redox status is critical for the maintenance of juvenility (Du et al., 2015). Glutathione redox status is determined on one hand by the total amount or concentration of free glutathione, on the other hand, by the GSH/GSSG ratio (Yu X. et al., 2013). It has been comprehensively summarized that the GSH/GSSG ratio is a critical environment for differentiation, proliferation, signal transduction, gene expression, and protein function (Ballatori et al., 2009). The severe drop of GSH and increased levels of GSSG in the chloroplast of apple hybrid trees might be caused by the progressively elevated H_2_O_2_ levels during the vegetative phase change (Jia et al., 2017). The great increase in relative levels of free glutathione, GSH, GSSG and the GSH/GSSG ratio in mitochondria can be explained by the adult phase elevated photorespiration and secondary metabolism, which produce more ROS and require more anti-oxidants (Cao et al., 2011; Gao et al., 2014). In the nuclei, intensive declines in the content of free glutathione, GSH, and GSSG as well the GSH/GSSG ratio were observed; this can be explained by the increased protein conjugated glutathione levels (Figure 2). The protein S-glutathionylation induced by changes in the GSH/GSSG ratio provides a thiol redox-sensitive signaling mechanism in the cell (Ballatori et al., 2009; Hill and Bhatnagar, 2012; Womersley and Uys, 2016).

### Phase-Related Subcellular Glutathione Depletion Is Associated With the Reduction of Soluble GGT Activity

Previously, we reported that the activities of glutathione reductase, ascorbate peroxidase, catalase, and monodehydroascorbate reductase were higher in the adult phases, which is inconsistent with the changes in GSH content in apple (Du et al., 2015). The age-associated declines in GSH levels and GSH/GSSG ratios in mammalians were caused by both the reduced GCL activity and the increased GSH consumption owing to the increased ROS generation in mitochondria or endoplasmic reticulum (Moor et al., 2014). The high levels of free glutathione partitioning in mitochondria in the adult reproductive phase in apple were consistent with mammalians, but GCL activity increased during the vegetative phase change (Figure 4B). The H_2_O_2_ accumulated in the chloroplast might have direct contributions to the elevated GCL activity (Preuss et al., 2014; Jia et al., 2017). Although GCL activity increased, the Cys content was significantly lower in the adult reproductive phase; this could possibly be due to lower levels of Cys recycling restricted by GGT activity or a deficit in the *de novo* biosynthesis of Cys.

In humans, glutathione declines with age due to the slower body protein turnover and thus lower availability of Cys and Gly, not the capacity to synthesize them (Sekhar et al., 2011). The activity of soluble GGT was significantly lower in the adult reproductive phase than that in the juvenile phase in apple. The enzyme GGT recycles Cys from GSSG and its conjugates, maintaining GSH/Cys homeostasis. GGT deficiency results in oxidative stress (Zhang and Forman, 2009). One of the *de novo* enzymes synthesizing Cys, CS, did not vary in the catalytic activity during the vegetative phase change in this study, but the activity of another enzyme synthesizing Cys, SAT, showed a drastic decrease with phase change, which might cause the reduction in Cys availability (Leustek et al., 2000). With the presence of acivicin, SAT activity and expression of *MdSAT1* and *MdSAT2* increased significantly in apple shoots *in vitro*, but the contents of free glutathione, and GSH decreased. The increased SAT activity could not compensate for the decrease in soluble GGT activity, which negated the upstream regulation of miR156 transcription by Cys biosynthesis but supported the dominant role of soluble GGT activity in the ontogenetic regulation of the cellular GSH buffering pool.

The apoplast redox status impacts GGT activity. In *Arabidopsis*, GGT1 becomes activated when the apoplast environment shifts to oxidative, and the GGT1 activity can be repressed by a more reductive apoplast environment (Zhang and Forman, 2009). The redox status was more reductive in the adult phase of apple hybrid trees because NADPH oxidase activity and the expressions of most *MdRboh* genes were significantly lower (Jia et al., 2017). In apple shoot apexes and leaves, *MdGGT* gene members, especially *MdGGT1*, were down-regulated in the adult reproductive phase; therefore, the decrease in soluble GGT activity could be attributed at least partly to transcriptional regulation (Figure 6).

### Soluble GGT Activity Regulated miR156 Precursor Gene Expression

OEMdMIR156/MIM156 did not affect soluble GGT enzyme activity or *MdGGT1* expression in transgenic apple shoots, indicating that the ontogenetic variation in miR156 is not an upstream regulator of GGT activity and *MdGGT1* expression. Like in *Arabidopsis*, the *ggt1* mutant is smaller in plant size and flowers sooner than the wild type, which exhibits sevenfold higher levels of GSSG and thus a low GSH/GSSG ratio (Ohkama-Ohtsu et al., 2007). Inhibition of GGT enzyme activity by exogenous reagents, SEB or acivicin, or transgenic methods reduced the free glutathione and GSH level and consequently suppressed the expression levels of *MdMIR156a5*, *MdMIR156a12*, and mature miR156. Conversely, overexpression of the *MdGGT1* gene could increase the levels of free glutathione and GSH, so the expressions of *MdMIR156a5*, *MdMIR156a12*, and miR156 were obviously activated.

GSH has a positive effect on adventitious rooting in tomato (*Lycopersicon esculentum* Mill) (Tyburski and Tretyn, 2010). Even in an auxin depleted condition, 200 μM exogenous GSH induces significantly higher number and length of adventitious roots in cucumber (*Cucumis sativus* L.) (Jiang et al., 2019). The potential of rooting is much higher in leafy cuttings from J or rejuvenated than A in apple (Xiao et al., 2014; Xu et al., 2017). As expected, the adventitious rooting ability was enhanced greatly in OEMdGGT1 apple transformants (Figure 10). Unlike SEB and acivicin, DEM forms DEM-GSH adduct, prevents GSH nuclear recruitment and impairs cell proliferation without interfering GGT activity (Vivancos et al., 2010). Depletion of glutathione levels by DEM induces a marked decrease in nuclear glutathione levels (García-Giménez et al., 2013). In this study, actually, depletion of GSH by using DEM treatment also caused a dramatic decrease in the expression levels of *MdMIR156a5*, *MdMIR156a12*, and mature miR156. GGT may affect miR156 level indirectly, via decreasing subcellular GSH level. An unexpected increase in *MdMIR156a5* and *MdMIR156a12* expression was found 1 h after DEM treatment, but mature miR156 level decreased significantly already at that time point.

It has been established that plant MIR156 transcription is under epigenetic regulation, such as H2A.Z deposition, H3K4, or H3K27 methylation (Xu M. et al., 2016; Xu Y. et al., 2016; Hyun et al., 2017; Xu et al., 2018). Oxidative stress inhibits histone demethylation and increases protein S-glutathionylation via changes in the GSH/GSSG ratio rather than in the intracellular H_2_O_2_ level (Hill and Bhatnagar, 2012; Niu et al., 2015; Womersley and Uys, 2016). GSH and the GSH/GSSG ratio therefore come to the forefront as redox regulation of vegetative phase change in higher plants. The potential mechanism remains as GSH increases the binding of transcription factors to the promoter of their target genes, GSH enhances the mRNA stability of some genes and/or some proteins can be S-glutathionylated on Cys residuals (Datta et al., 2015).

## Data Availability

This manuscript contains previously unpublished data. The name of the repository and accession number are not available.

## Author Contributions

XZ, ZZ, YW, TW, ZH, and YC prepared the plant materials and designed the experiments. YC, QZ, KC, and XJ conducted the experiments. YC and QZ took the photographs. YC, QZ, and XJ analyzed the data. YC wrote the manuscript. All authors read and approved the manuscript.

## Conflict of Interest Statement

The authors declare that the research was conducted in the absence of any commercial or financial relationships that could be construed as a potential conflict of interest.

## Footnotes

1 https://www.rosaceae.org/

2 http://www.ncbi.nlm.nih.gov/genbank/

3 http://www.takara-bio.com/

## Abbreviations

Acivcin: α-amino -3-chloro-4,5-dihydro-5-isoxazoleacetic acid
A: adult phase
Cys: cysteine
CS: cysteine synthase
DEM: diethyl maleate
GSH: reduced glutathione
GSH/GSSG: glutathione/glutathione disulfide
GGT: Gamma ( γ )-glutamyl transpeptidase
GCL: glutamate-cysteine ligase
J: juvenile phase
LSD: least significant difference
MIM156: miR156-mimetic
miR156: microRNA156
ROS: reactive oxygen species
SAT: serine acetyltransferase
SEB: serine-borate complex.
