# Supplementary material for: MdGGT1 Impacts Apple miR156 Precursor Levels via Ontogenetic Changes in Subcellular Glutathione Homeostasis
Source: Front Plant Sci. 2019 Jul 31;10:994. doi: 10.3389/fpls.2019.00994 (PMC6684775; doi:10.3389/fpls.2019.00994)
Supplement: Supplementary file 1 [file Table_1.DOC]

**Supplementary Figure**

**
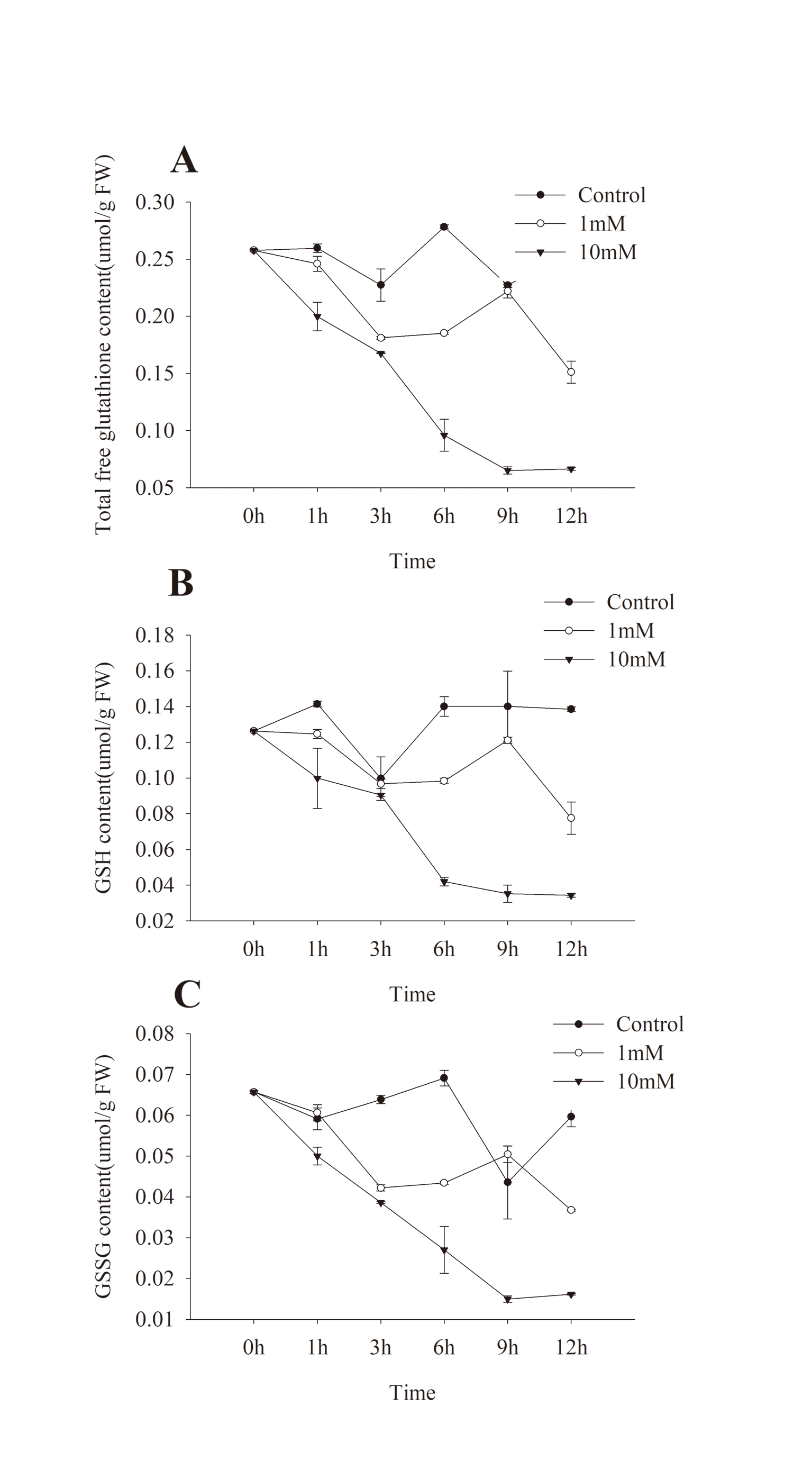
**

**Fig S1.** Dynamic changes in total free glutathione (A) , GSH (B) and GSSG (C) contents in apple *in vitro* shoots with or without 1 uM or 10 uM diethyl maleate (DEM) added to the culture media. Error bars represent the standard deviations of three experimental replicates.

**
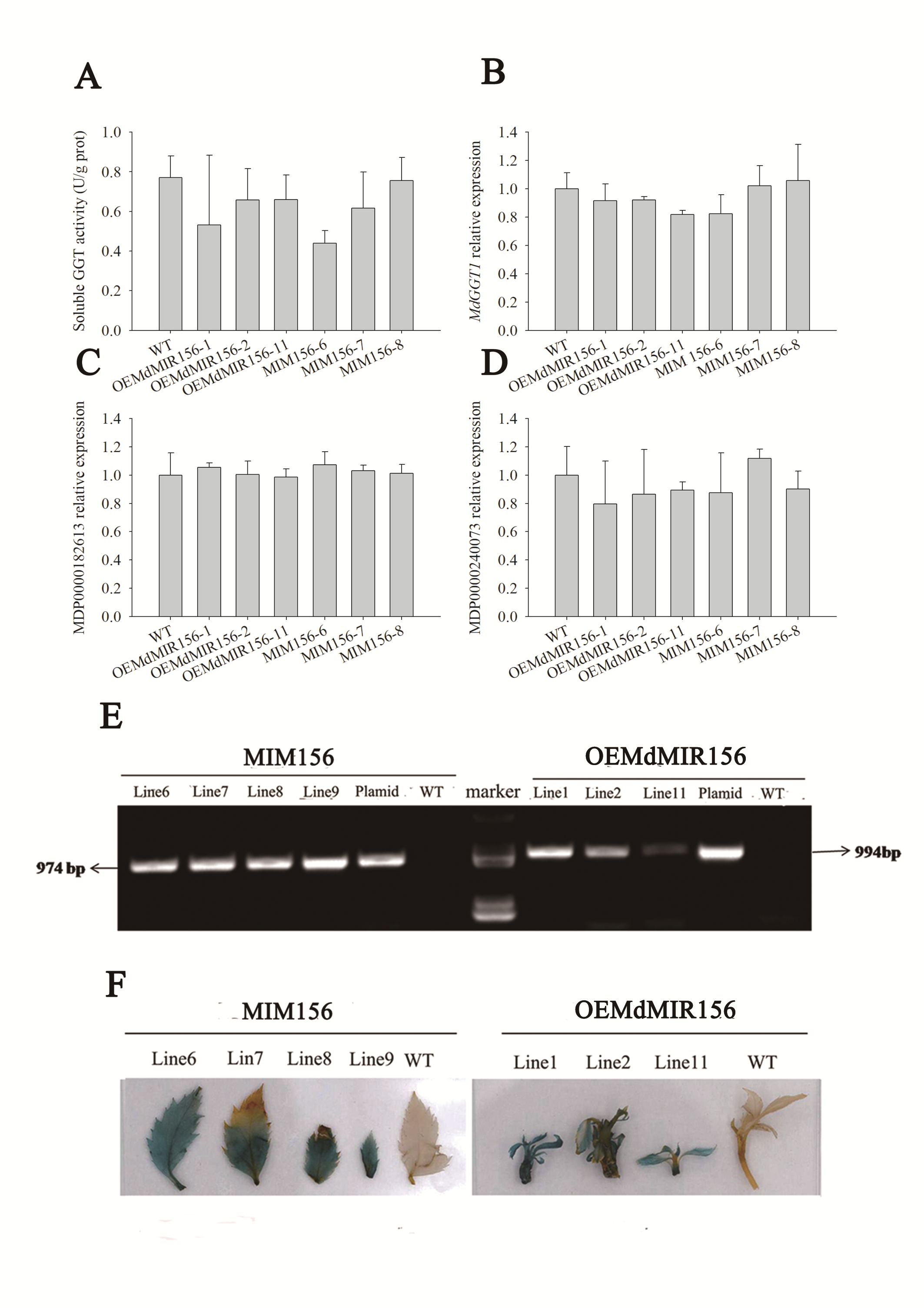
**

**Fig S2.** Soluble GGT activity (A) and relative expressions of *MdGGT1*, MDP0000182613 and MDP0000240073 (B, C, D) in OEMdMIR156a6 and MIM156 transgenic apple (GL-3) lines. Panel E shows the PCR products of 974 bp 35S::MIM156-GUS (left) and 994 bp 35S::MdMIR156a6-GUS (right) constructs (GUS was used as reporter gene) identified in OEMdMIR156a6 and MIM156 apple transgenic lines. Panel F shows the GUS histochemical staining of OEMdMIR156a6 and MIM156 apple transgenic lines. Error bars in panel A~D represent the standard deviations of six experimental replicates.


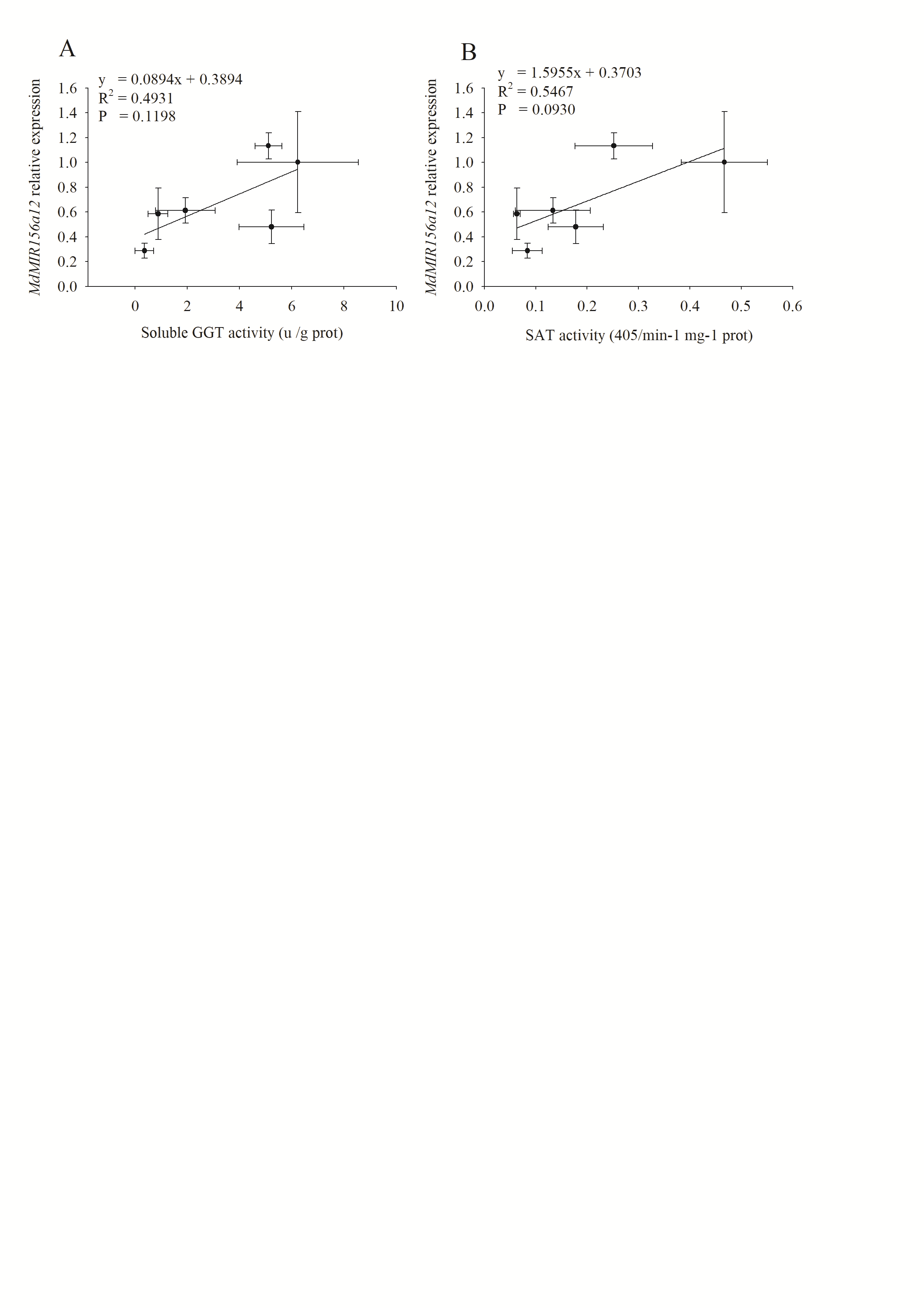


**Fig S3.** Correlations between relative expressions *MdMIR156a12* and the activity of soluble GGT and SAT in leaves during ontogenesis of apple hybrids (*Malus asiatica ‘*Zisai Pearl’ × *M. domestica ‘*Red Fuji’).

**Fig S4.** Alignment between DNA sequence of *AtGGT1* and apple MDP0000319231 gene by Sanger sequencing

**Fig S5.** Alignment between DNA sequence of *AtGGT2* and apple MDP0000319231 gene by Sanger sequencing

**
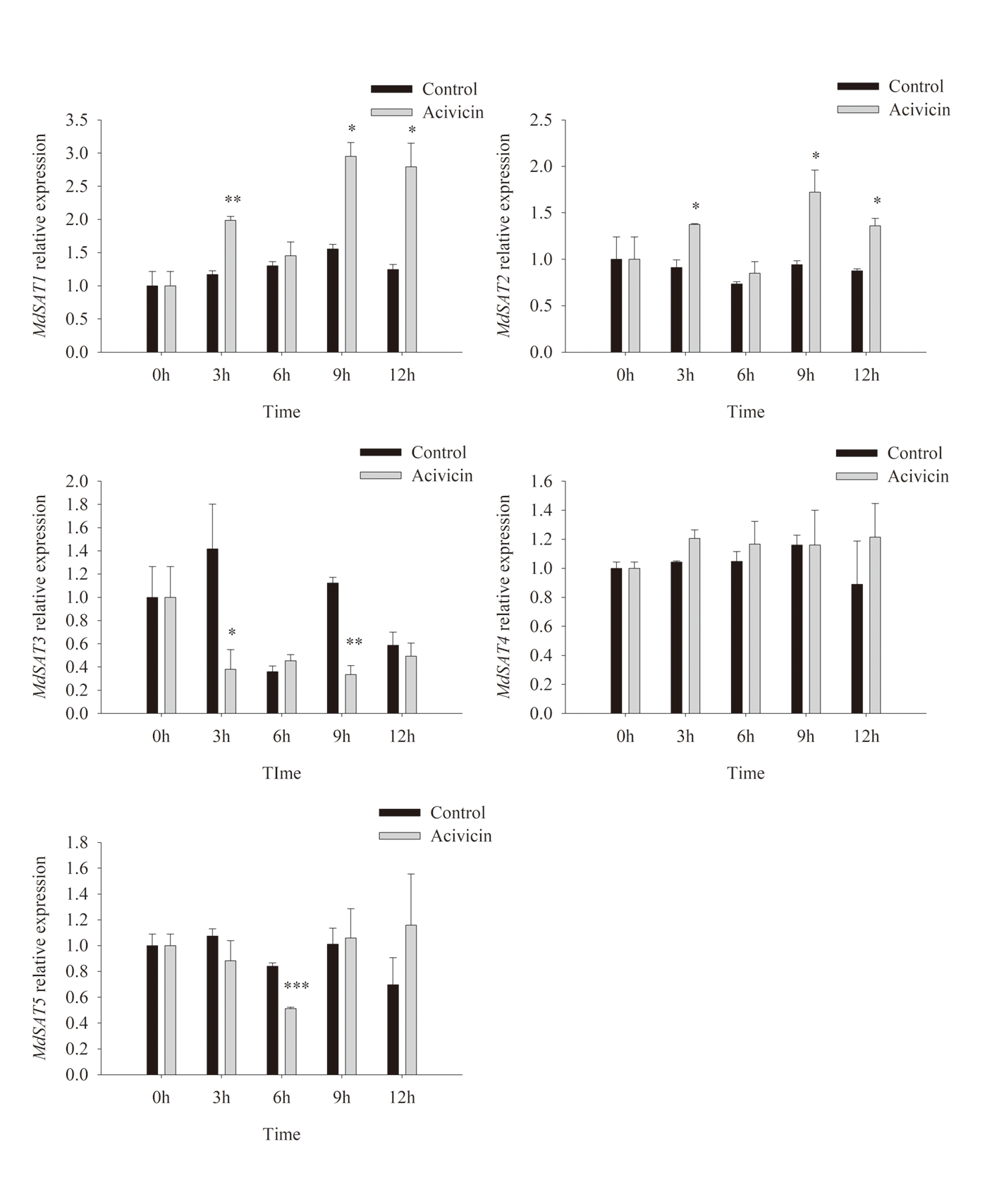
**

**Fig S6.** Changes in relative expressions of *MdSAT* gene members in apple *in vitro* shoots with or without addition of 50 uM α-amino-3-chloro-4, 5-dihydro-5-isoxazoleacetic acid (acivcin) to the culture media. Error bars represent the standard deviation of three biological replicates. “*”, “**” and “***” indicate significant difference at P < 0.05, P < 0.01 and P < 0.001, respectively.


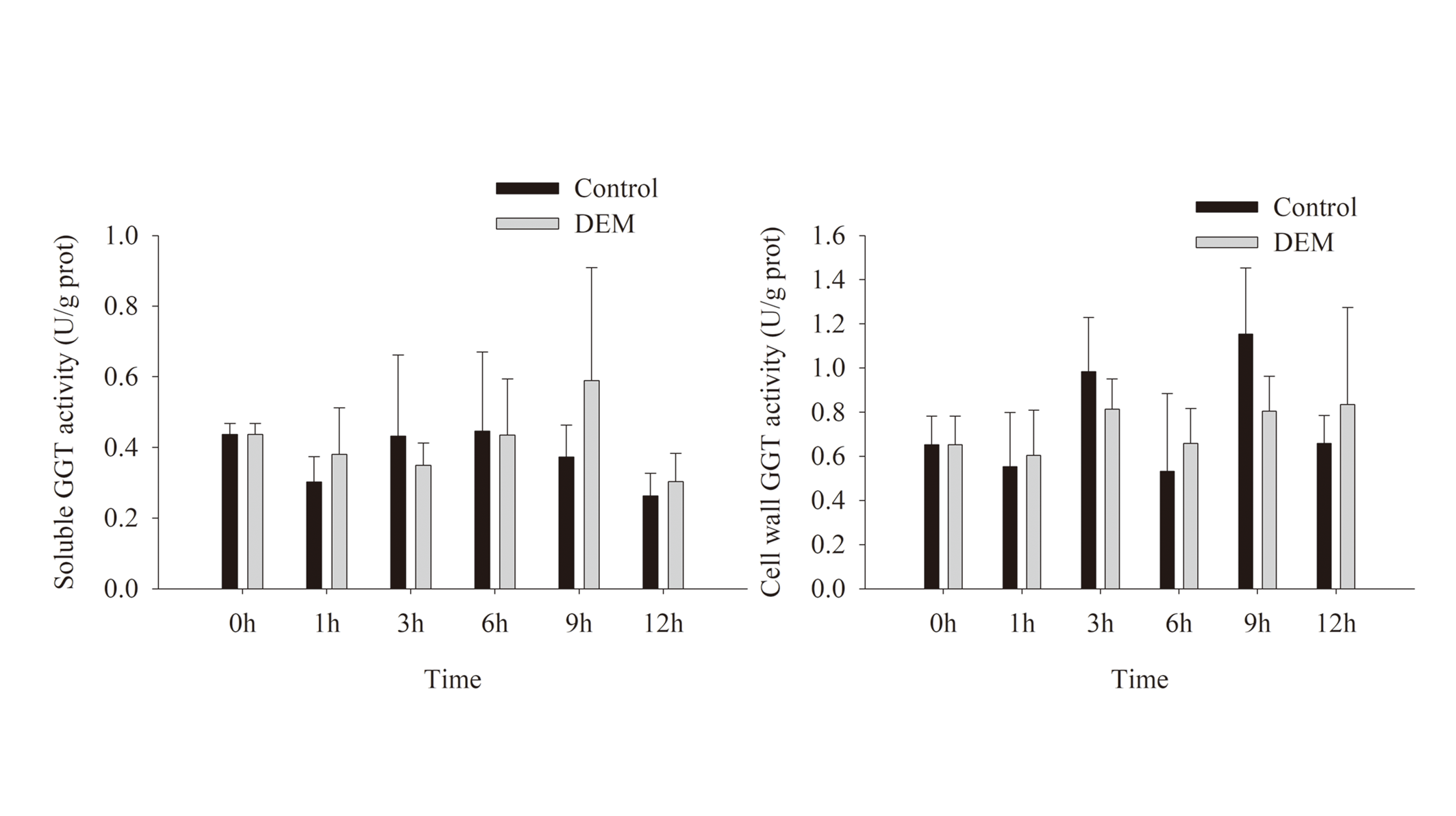


**Fig S7.** Changes in enzyme activities of soluble and cell wall conjugated GGTs in apple *in vitro* shoots with or without addition of 10 uM diethyl maleate (DEM) to the culture media. Error bars represent the standard deviation of three biological replicates.
