# Supplementary material for: MdGGT1 Impacts Apple miR156 Precursor Levels via Ontogenetic Changes in Subcellular Glutathione Homeostasis
Source: Front Plant Sci. 2019 Jul 31;10:994. doi: 10.3389/fpls.2019.00994 (PMC6684775; doi:10.3389/fpls.2019.00994)
Supplement: Supplementary file 2 [file Table_2.DOC]

| **Table S1.** Primers for the eight full-length *MdGGT* genes | |
| --- | --- |
| Primer name^a^ | Primer sequence |
| MDP0000319231_F | 5’-ATGGGGGAGCAGAGCTTGGAA-3’ |
| MDP0000319231_R | 5’-TCATACGGCTGCAGGCC-3’ |
| MDP0000182613_F | 5’-ATGGGGCAGCAGAGCTCGGAAG-3’ |
| MDP0000182613_R | 5’-TCATACAGCTGCAGGCCTCCCGT-3’ |
| MDP0000240073_F | 5’-ATGGGTGCTTTTAACCCAACGGATA-3’ |
| MDP0000240073_R | 5’-TCATACAGCTGCGGGCCTCCCGTCC-3’ |
| MDP0000146709_F | 5’-ATGGGAAATTCTGATTGGATGATG-3’ |
| MDP0000146709_R | 5’-TTAGAAACCAGCCGGAACCC-3’ |
| MDP0000239530_F | 5’-ATGGGGCAGCAGAGCTCGGAAG-3’ |
| MDP0000239530_R | 5’-TCATACAGCTGCAGGCTTCCCGTC-3’ |
| MDP0000278877_F | 5’-ATGCTACGAGCGGTTTCACCGAAAG-3’ |
| MDP0000278877_R | 5’-TTAAAGGACCGTTTGGATTATCTCT-3’ |
| MDP0000285996_F | 5’-ATGCTGCTGTTGCCACATGATCTTC-3’ |
| MDP0000285996_R | 5’-TCATACGGTTGATGGAGCAGATTG-3’ |
| MDP0000122801_F | 5’-ATGCTACGAGCGGTTTCAC-3’ |
| MDP0000122801_R | 5’-TTAAAGGACCGTTTGGATTATCT-3’ |
| ^a^ F, forward; R, reverse. |  |

| **Table S2.** Primers for qRT-PCR |  |
| --- | --- |
| Primer name^a^ | Primer sequence |
| qMDP0000319231_F | 5’-TCTACCACAGGCTGATACCG-3’ |
| qMDP0000319231_R | 5’-GCATCTTATGACCTCGCTCT-3’ |
| qMDP0000240073_F | 5’-AAGCGGAAAGAACTCTAATG-3’ |
| qMDP0000240073_R | 5’-GCAAGACTAAATATCCCACAA-3’ |
| qMDP0000182613_F | 5’-TGTAAGTGACCCGAGAAAGGA-3’ |
| qMDP0000182613_R | 5’-ATTGTGGAAATTTATGTAACATTTG-3’ |
| qMDP0000239530_F | 5’-ACATAGGCCGGAAAGGC-3’ |
| qMDP0000239530_R | 5’-TAAATATCCCACAAGGCACTC-3’ |
| β-Actin_F | 5’-TGGTGAGGCTCTATTCCAAC-3’ |
| β-Actin_R | 5’-TGGCATATACTCTGGAGGCT-3’ |
| qmiR156_F | 5’-TGCACTAGCGTGTGACAGAAGA-3’ |
| qmiR156_R | 5’-ACATCGTATCGTGAAG-3’ |
| 5sRNA_F | 5’-TGCACTAGCGTGTAGAGGAACC-3’ |
| 5sRNA_R | 5’-ACATCGTATCGTGAAG-3’ |
| qMdMIR156a5_F | 5’-AGCTGACGGAGAGAGAAGT-3’ |
| qMdMIR156a5_R | 5’-CTGAGCGTAGTTGACAGAAGAG-3’ |
| qMdMIR156a12_F | 5’-CACGCAAAGGTATGGCTTTATAC-3’ |
| qMdMIR156a12_R | 5’-GCAGAGAGAGAGAAGGTGAATG-3’ |
| qMdSAT1_F | 5’-GGCGAGAAAGTTAGAAGAGGAG-3’ |
| qMdSAT1_R | 5’-AGAAGCTCAGCATCAGCATAG-3’ |
| qMdSAT2_F | 5’-CCTCCTACTAGTTCAGCCATTTC-3’ |
| qMdSAT2_R | 5’-GGTGCGAGACAGTTCTCTTTAT-3’ |
| qMdSAT3_F | 5’-GCTACTCTGATGGTTGCTAGAATA-3’ |
| qMdSAT3_R | 5’-TAAGGCTGTCTCGGAATAAACC-3’ |
| qMdSAT4_F | 5’-AGCTACCTCTACTCCACCATAC-3’ |
| qMdSAT4_R | 5’-TGTTGAGGAAGAGGTCGTAGA-3’ |
| qMdSAT5_F | 5’-GTCAGTGGTGCTTATCGATGT-3’ |
| qMdSAT5_R | 5’-TGGCACATCCTCATGTTTAGAG-3’ |
| ^a^ F, forward; R, reverse. |  |

| **Table S3.** Primers for reverse transcription of miR156 and 5sRNA | |
| --- | --- |
| Primer name | Sequence (5′→3′) |
| RT-miR156 | GTCACATCGTATCGTGAAGCTGCGCAGCTGATGTGACGTGCTCAC |
| RT-5s rRNA | GTCACATCGTATCGTGAAGCTGCGCAGCTGATGTGACTGGATTGG |

| **Table S4.** Primers for construction of vector | |
| --- | --- |
| Primer name^a^ | Primer sequence (5′→3′) |
| OEGGT1_F | TCCCCCGGGATGGGGGAGCAGAGCT |
| OEGGT1_R | CGGGATCCTCATACGGCTGCAGGCC |
| GGT1forward_F | GCTCTAGAAAGACTGCCAGACCTCTCT |
| GGT1forward_R | TCCCCGGGGGTTTGGGAGGTTGCTGA |
| GGT1reverse_F | CGAGCTCAAGACTGCCAGACCTCTCT |
| GGT1reverse_R | GGGGTACCGGTTTGGGAGGTTGCTGA |
| ^a^ F, forward; R, reverse. | |

| **Table S5.** Primers of DNA-PCR identification | |
| --- | --- |
| Primer name^a^ | Primer sequence (5′→3′) |
| OEMdGGT1_F | CTATCCTTCGCAAGACCCTTC |
| OEMdGGT1_R | CAGGAAACAGCTATGAC |
| RNAiGGT1_F | GCTCTAGAAAGACTGCCAGACCTCTCT |
| RNAiGGT1_R | CAGGAAACAGCTATGAC |
| OEMdMIR156_F | GACGCACAATCCCACTATCC |
| OEMdMIR156_R | TTTCCCACCAACGCTGATC |
| MIM156_F | GACGCACAATCCCACTATCC |
| MIM156_R | TTTCCCACCAACGCTGATC |
| ^a^ F, forward; R, reverse. | |

**Table S6**. Adventitious rooting ability of transgenic apple lines over expressing MdGGT1 and MdGGT1-RNAi. Means ± SE, n = 4. The different lower-case letters shown on the third column indicate the statistical significance (p < 0.05) by analysis of variance followed by Duncan’s multiple-range test.

| Materials | Rooting rate (%) | Number of roots per plant |
| --- | --- | --- |
| Wild type | 5±2.5 | 0.25±0.125 b |
| OEMdGGT1 | 7.5±1.25 | 2.75±0.515 a |
| RNAiGGT1 | 0 | 0 c |
